# Health and social care services for people with dementia at home at the end of life: A qualitative study of bereaved informal caregivers’ experiences

**DOI:** 10.1177/02692163221092624

**Published:** 2022-04-23

**Authors:** Caroline Mogan, Karen Harrison Dening, Christopher Dowrick, Mari Lloyd-Williams

**Affiliations:** 1 Academic Palliative and Supportive Care Studies Group (APSCSG), Primary Care and Mental Health, University of Liverpool, Liverpool, UK; 2 Dementia UK, Research and Evaluation, London, UK

**Keywords:** Dementia, end of life, informal care, relatives, palliative care, qualitative study

## Abstract

**Background:**

More people are dying at home with dementia and Alzheimer’s disease. While informal caregivers are the main providers of care for people with dementia dying at home, they require support from health and social care services. However, little is known about how they experience these services.

**Aim:**

To explore informal caregivers’ views and experiences of health and social care services when looking after a person with dementia at home at the end-of-life.

**Design:**

A qualitative interview study. Data were analysed using thematic analysis.

**Setting/Participants:**

Twenty-nine bereaved informal caregivers who had looked after a person with dementia at home during the last 6 months of life.

**Results:**

Specialist palliative care for people with dementia dying at home is rare and care is mostly managed by General Practitioners and domiciliary care workers. Four overarching themes were identified: Poor continuity of care; Lack of expertise; Limited advance care planning; and Loss of autonomy.

**Conclusions:**

End-of-life care at home for people with dementia must be proactively planned with an emphasis on advance care planning. Policy makers should recognise the critical role of domiciliary care services in end-of-life care and ensure that they are adequately qualified and trained.

**What is already known about the topic?**

- More people are dying of dementia within the family home.
- Informal caregivers play a crucial role in delivering care for people with dementia at home at the end-of-life, yet little is understood about their experiences.
- A palliative approach to care is recommended across all stages of dementia. However, it is often only started in the advanced stages of the disease, if at all.

**What this paper adds?**

- Specialist palliative care for people with dementia living in their own home is rare and mostly managed by GPs and domiciliary care workers. However, informal caregivers question their expertise in dementia and end-of-life care.
- Domiciliary care was reported to be inflexible, with an over-emphasis on a task-centred approach resulting in care that lacks consideration of individual wishes and circumstances.
- Discussions with health and social care professionals about end-of-life wishes and the formal documentation of preferred place of care is largely absent.

**Implications for practice and policy**

- Informal caregivers providing end-of-life care for people with dementia at home urgently require good quality, affordable domiciliary care services.
- They should also have access to competent support and assistance 24 h a day, as well as clear advice on how to obtain help in an emergency.
- People with dementia should be offered the opportunity to consider their preferences for end-of-life care through the process of advance care planning early in the disease trajectory, this includes having choice over where death occurs.

## Introduction

Dementia is a life-limiting illness that is associated with a high symptom burden, particularly in the advanced stages of the disease.^1^ By 2040, it is estimated that 220,000 people will die each year with dementia in England and Wales, and many will have palliative care needs.^2^ Owing to this increase in prevalence, providing appropriate end-of-life care to people with dementia now poses a significant challenge to health care systems, families and societies across the world.

Place of death is identified as an important component of high-quality end-of-life care and it is reported that most people would prefer to die at home.^3–5^ In the UK, the number of deaths in private homes has been generally increasing since 2005, but in 2020 there was a large increase (29.2% higher than 2019).^6^ While Ischaemic heart diseases were the leading cause of death in private homes, dementia and Alzheimer’s disease saw the largest increase in deaths compared to the 5-year average; 72.5% and 60.7% increase for males and females respectively (1433 and 2485 more deaths).^6^

The significant increase in the number of home deaths since the start of the COVID-19 pandemic is likely to have been driven by a combination of factors including health service disruption, people choosing to stay away from health care settings, or terminally ill people staying at home rather than being admitted to hospital for end-of-life care. This is supported by Hanna et al.^7^ who reported that even after the easing of public health restrictions, people with dementia were fearful of re-entering society through concerns of contracting or spreading COVID-19.

The possibility of being cared for at home through to the end-of-life is largely dependent upon informal caregivers (referred to herein as ‘caregivers’) who can provide a substantial part of the caregiving.^8,9^ However, as a person’s dementia progresses, they typically have more extensive needs than their caregivers can provide and become increasingly reliant on assistance from health and social care professionals.^10^

In the UK, there is now a greater emphasis on supporting people with dementia in the community with a focus around Memory Services, which are secondary care services that assess, diagnose and treat dementia. Memory Services are often multidisciplinary, consisting of old age psychiatrists, mental health nurses, occupational therapists, social workers and psychologists. Teams may also have input from physiotherapists, pharmacists, speech and language therapists and support workers. The composition varies depending on historical and local needs, and there is currently no agreed national standard for staffing or length of input.^11^

Social care in the UK can take several different forms depending on the needs of the person. Examples include day care, respite and domiciliary care. Unlike health care services that are ‘free at the point of delivery’ with funding provided to the NHS directly by the central government, funding for adult social care is from multiple sources and the responsibility of local authorities. Consequently, individuals who wish or need to use social care services are subject to means-testing to determine if they are eligible. Only those deemed to have very high needs receive full public-funded support, with most having to pay for some or all of their social care.

It is recommended that a palliative approach to care is provided across all stages of dementia.^12,13^ However, despite the range of services involved over the course of the dementia trajectory, it is often only started following a crisis or when dying is believed to be imminent.^14^ The marked increase in dementia deaths at home has notable implications for health and social care resources. Exploration of caregiver experiences offers valuable insights into how professional services provide care and support to families at the end-of-life. This will aid our understanding about how they can best be equipped in the future.

## Aim

The aim of this study is to explore how caregivers experience health and social care services when providing end of life care at home for a person with dementia.

## Methods

### Design

This was a descriptive qualitative study based on a constructivist epistemology using in-depth semi-structured interviews. Interviews aimed to explore participants’ experiences of caring for a person with dementia at home at the end of life, and their engagement with health and social care professionals.

### Participants

Using purposive sampling techniques, bereaved informal caregivers who had looked after someone with dementia at home during a substantial part of their last 6 months of life were recruited. Due to the sensitive nature of the topic and the potential for distress, those bereaved within the last 3 months were excluded from participation. Additionally, those who were bereaved longer than 3 years were also excluded as it was felt that this may affect recall.

### Recruitment

A broad approach to recruitment was used. Appeals for participants were made via posters displayed in public venues (e.g. GP practices and community halls). Additionally, the National Institute for Health Research’s ‘Join Dementia Research’ was used; an online self-registration service, that enables volunteers to register their interest in taking part in dementia research.

Participants were also identified and approached by clinicians known to them from local Memory Services. After being given an information pack, the participant could then contact the research team directly or return a reply slip to express an interest in taking part.

### Data collection

Interviews were conducted between November 2017 and June 2018 by one of the authors (C.M.), an experienced dementia researcher. An interview schedule was developed, informed by an earlier review,^15^ and the research team, consisting of clinicians and researchers in primary, dementia and palliative care. This was iteratively modified throughout the data collection period to ensure follow-up with categories in subsequent interviews.

All interviews were audio-recorded. Most took place in the participants own homes, often where the person with dementia had died, which provided a sense of place that also helped to inform the interpretation of the data. Two took place on university premises and one via Skype. Extensive field notes were taken following each interview.

### Data analysis

Interviews were transcribed *verbatim*, anonymised and analysed thematically by C.M. NVivo 11 (QSR International (UK) Ltd) was used to organise data. Transcripts were read, re-read and coded inductively for themes relevant to participants’ experiences and perceptions of health and social care services. A coding frame was developed and checked against the data to ensure fit. 10% of transcripts were double coded by another researcher (M.L.W.), who independently produced a coding frame which was triangulated with the main framework. The coding framework was reviewed, and related codes were grouped into themes. Themes were defined and finalised through discussion, and all authors agreed with the final analysis, interpretation and reporting.

### Ethical considerations

Participants were provided with oral and written information about the study and provided written informed consent. They were informed they could stop the interview at any time due to its sensitive topic, however, none expressed a wish to stop an interview or required additional support. Though research participants affected by serious illness can find research interviews to be a positive experience,^16,17^ participants were provided with information about support organisations as part of the study debrief.

Ethical approvals were obtained from University of Liverpool Central University Research Ethics Committee [Ref: 1392].

## Results

Twenty-six interviews were conducted with 29 bereaved caregivers. This included 23 individual interviews, and three interviews with two participants, who asked to be interviewed together. Most participants were female (n = 23) and predominantly daughters of the person with dementia (n = 12). Sample characteristics are reported in Table 1.

**Table 1.** Characteristics of caregiver and person with dementia (PwD).

Variable	*N*
Gender	
Female	23
Male	6
Age of caregiver	
20–29	1
30–39	0
40–49	2
50–59	7
60–69	10
70–79	5
80–89	4
Age of PwD at death	
60–69	2
70–79	5
80–89	11
90–99	8
Relationship to PwD	
Spouse/partner	9
Female (*n* = 6)	
Male (*n* = 3)	
Adult child	14
Son (*n* = 2)	
Daughter (*n* = 12)	
Granddaughter	1
Niece	3
Friend	2
Female (*n* = 1)	
Male (*n* = 1)	
Years spent caring	
0–5	16
6–10	11
11–15	2
Place of death	
Home	16
Hospital	2
Care home	7
Hospice	1

The data below is representative of caregivers’ experience of health and social care services when providing care to a person with dementia at home at the end-of-life. All participants received community-based health and social care from a range of professionals. Four overarching themes were identified: Poor continuity of care; Lack of expertise; Limited advance care planning (ACP); and Loss of autonomy.

### Theme 1: Poor continuity of care

Continuity of care occurs when healthcare events are experienced as coherent, connected and consistent.^18^ Caregivers described a significant impact on their experiences of caring at home as a result of the lack of *relational continuity* (An ongoing patient-professional relationship), *management continuity* (consistent and timely coordination of care and services) and *informational continuity* (the effective transfer and use of patients’ past and current personal information).

*Relational Continuity –* Caregivers believed that an ongoing relationship with professionals was important due to a desire that they would have previous knowledge of the person with dementia. This was significant within the context of domiciliary care, as caregivers felt it was essential in upholding the dignity of the person with dementia. Having the same care workers attending to personal care had the effect of changing the relationship from ‘stranger’ to someone who was trusted. While it was acknowledged that it would be difficult for the same care workers to visit each time, caregivers felt that having many different ones was concerning, especially when they were assisting with personal care:

*There were so many coming in as well and I used to think sometimes for personal care it would be nicer to have a small team rather than a lot of people coming in to sort out her toileting and whatever, in an ideal world that would be great but I understand it’s not, and having somebody to cover the rota is the best you could hope for (Participant 16, Daughter)*

*Management Continuity –* Caregivers felt that ongoing monitoring of physical and mental health was important. Many felt that this should be the role of Memory Services. However, it was evident that there was significant variation in the support that they received and the length of time they were followed up. Some were seen only for diagnosis:

*We asked for another appointment at memory clinic but the memory clinic only diagnosed* *us, there was absolutely no help given to us, we were given a few leaflets but nothing really of any help (Participant 07, Daughter)*

Others reported that despite being well supported through the earlier stages of the disease, as soon as the person reached the advanced stages, they were discharged. Some believed that this was because Memory Services had limited knowledge about palliative care and end-of-life issues:

*The mental health nurse when she was involved, she was quite good but actually she withdrew, probably five months before mum died it was almost as if it was out of her remit really as to what to do next, she was out of her knowledge and depth as to what to do to keep mum at home (Participant 04, Daughter)*

Some caregivers felt well supported by Memory Services through all stages of the dementia trajectory, and they were able to gain advice about end-of-life care issues, which helped them feel more prepared to care at home. One described their mental health nurse from the Memory Clinic as a ‘lifeline’ as the end-of-life drew closer as she was knowledgeable and easy to contact:

*The [Memory Clinic] nurse that came here was really a lifeline to me, so she was really good because she, although she came round every three months, six months, I could always make an appointment before then if I needed to but she was really good to talk to and I could say “[name of nurse] what happens at the next stage, what are we going to do” and at the last stage she had already warned me because I’d asked her “what do we do when I can’t get her out of bed anymore” and she said she’ll have to get onto the GP who will have to sort out care at home or will get the nurse to come round so I always knew that that would happen (Participant 03, daughter)*

Along with issues with regular monitoring, there was also a perception of disorganisation during times of crisis. Crisis was usually defined as a period of deterioration or ill health in the person with dementia or in themselves (or another person closely involved in the persons care), or when the person with dementia had a fall. Not knowing who to call in the event of a crisis meant that many relied upon emergency services:

*We called a lot of ambulances too, they were super, they were very kind and understanding, nothing was too much trouble. . .when she collapsed one night in the bedroom I did 999 but I said “it’s not a blue light job but I need assistance to get her downstairs and just check over her but as far as I can see she’s in pain and I think it might be her rib”. . .so I got [my wife] comfy and came downstairs and I put the downstairs light on and I thought “bloody hell, it’s a blue light”, they’d picked the call up and they were here so it was a super service and they were very kind (Participant 011a, Husband)*

Other participants believed this to be a misuse of resources. Additionally, a call to emergency services often meant that the person with dementia was admitted to hospital, even when it was felt this was not required. This could result in lengthy stays, causing detriment to the person’s health status.

*Information Continuity –* Poor information exchange between health and social care services could also result in the person with dementia receiving inappropriate interventions. Examples were given when poor communication between services resulted in the person with dementia being given medications that were contraindicated. Another event was described when a GP did not notify the out of hours team to affirm that the person with dementia was on the end-of-life care pathway and had a Do not Attempt Cardiopulmonary Resuscitation (DNACPR) order in place. This resulted in the persons elderly husband being told to call the urgent health advice service (NHS 111), when finding his wife had died. Subsequently they advised giving her emergency cardiopulmonary resuscitation (CPR), with distressing repercussions:

*They start off with the “is the patient breathing” the flowchart, no ok, he’d forgot about the DNAR, he’d just found his wife dead and they said “right you need to get her out the bed onto the floor and start CPR”. . ..so he did, he dragged her out the bed, she had a catheter in place, dragged her out of the bed and started CPR. . .for ten minutes, by the time we got there the police were there, they could see it wasn’t anything untoward but because of the circumstances she had to go to the coroner, all because [the GP] didn’t notify the people he was meant to say that she was a DNAR and for that reason [my Grandad] did CPR (Participant 18a, Granddaughter)*

This experience tainted the family’s view of home death, with them stating that they regretted their decision to keep the person with dementia at home.

### Theme 2: Lack of expertise

Due to an absence of specialist palliative care for people with dementia, GP’s were often the main providers of information about end-of-life care at home. Despite their input, caregivers expressed concerns that GPs did not possess the necessary skills and knowledge about dementia:

*The GP was excellent although as he said himself “I don’t know anything about dementia, the word wasn’t mentioned during my course” and he was only in his 40’s you know and so he was learning on the job (Participant 25, Wife)*

Others felt that GP’s lacked judgement in end-of-life issues in general and had limited knowledge about palliative care:

*That plonker of a GP. . .this was the point when he wanted her to go into hospital, he said “well she’s not eating and drinking”. . .I said “because she’s deteriorating and approaching the end-of-life”, I felt that it was always me telling him, he didn’t have a clue and would have just put her in hospital and that’s where she would have died (Participant 09, Son)*

There were also serious concerns about domiciliary care workers lacking basic knowledge about issues related to dementia:

*I think that was one of my biggest bugbears with caring at home, is the carers, they need to be trained and they never are, I mean anybody, whatever age can go out and get a job as a carer they don’t need to be qualified, they don’t need experience, so none of them know how to handle and bath them because they don’t have any training, they don’t have any knowledge of dementia, they don’t seem to have any knowledge of old people, I think it’s disgusting, you wouldn’t let anyone who was unqualified look after your children so why would you want to let them look after your mum (Participant 08, Daughter)*

One professional group that did appear to be knowledgeable in both end-of-life and dementia issues were Admiral Nurses. However, only one participant had utilised this service. Their general perception was that the Admiral Nurse was knowledgeable about symptom control and available resources, had influence over other health professionals and got things done promptly. The nurse was also described as being emotionally supportive:

*At the same time we got the referral to the Admiral Nurse, they did a lot practically, a lot, but also emotionally as well because they got us to reflect back on what we’d achieved which you kind of forgot about that, you know, but it did remind you that you’re doing a good job here which tends to be missed a little bit (Participant 26a, Niece)*

In addition, this participant also explained that it was only when the Admiral Nurse had got involved that it seemed possible that the person with dementia could be cared for at home until the end, as all of the other services appeared to be advocating a care home placement.

### Theme 3: Limited ACP

Most caregivers were unaware of the process of ACP and did not recall documenting end-of-life plans with the person with dementia and health care professionals. However, most believed that talking about future care, for example in terms of treatments and place of care, was necessary. More specifically, they thought that the GP should discuss this and provide help with possible end-of-life decisions.

Some felt that end-of-life conversations with professionals should take place early on but often this was not the case:

*Participant: My dad made the decisions for her because he knew what she would have* *wanted but she never actually verbalised it because she wouldn’t with us as a family. . .but had there been a healthcare professional that had said to her “look, this is what’s happening and actually this is how things will deteriorate but we want to put some plans in place for you, what’s going to be important?” she may well have had some of that discussion when she was able to*

*Researcher: so who do you think would have been best placed for that*

*Participant: Probably the GP because it’s the GP you’ve known for a long time and it (Participant 04, Daughter)*

When discussing future planning of care, caregivers spoke more specifically about legal documents such as Lasting Power of Attorney (LPA) and DNACPR. Some recalled discussions with their GP about preferences for the location of end-of-life care but again they were not formally documented. Some stated that future planning of care had only come into fruition after conversations they had with friends or family who had been through similar experiences:

*When we did get to the stage when things were really bad we were phoning the doctors regularly but they were just dealing with the problem and not really assessing her properly so we ended up getting advice from a friend who said to ring the doctors and ask for a “Statement of Intent”, a fast tracking of Continuing Health Care and a “do not resuscitate” and it was only at that point when we said that that they started moving (Participant 26b, Niece)*

Overall, there was a sense that professionals were not proactive in having end-of-life discussions and caregivers had to seek out this information themselves.

### Theme 4: Loss of autonomy

A common source of tension amongst caregivers was a feeling that their lives were controlled by health and social care services. Many worried that professionals had the authority to ‘take away’ the person with dementia. This could often result in families being left in distressing situations because they had concealed the severity of their needs in case they had been perceived as being unable to cope. This was especially relevant for older participants as well as those who did not have an LPA:

*[Social Services] did their best for mum but because I hadn’t got the Lasting Power of Attorney I had to keep things going and play the game with [the Social Worker] because I was afraid that they would come along and I wouldn’t be able to stop them from taking her (Participant 15, Daughter)*

Others explained how care packages had been implemented quickly during times of crisis, without their consultation. Domiciliary services especially, were criticised for being too rigid in their approach with caregivers finding it frustrating that some tasks could not be swapped for others that might be more important on a given day. This did not seem to be a good way of tailoring care to the person’s needs:

*I mean some days he just needed a shave, or his nails cutting or something like that but they wouldn’t do it because it wasn’t on their list or it was against health and safety, sometimes I’d think “what am I even paying you for” (Participant 22, Wife)*

Others stated that the care provided was organised as a series of tasks that were rushed through. This contributed to a highly regimented environment, resulting in care that felt undignified:

*They used to rush her because in half an hour [the carers] would do stuff that I’d take over an hour to do, she’d be mauled about “drink this, have that, come on face washed, do this” it was too much (Participant 23, Daughter)*

How caregivers financed domiciliary care appeared to have a considerable impact on their feeling of autonomy. Care was often arranged following a local authority assessment and was provided by an agency and paid for in full either by the local authority or with a contribution from themselves. For these families there was a sense that they had little alternative but to take the care that was offered to them even if it did not meet their requirements.

Some participants had the financial resources to pay privately for care, either using this type of care exclusively or ‘topping up’ on local authority funded care with private carers. These participants usually described a better experience of domiciliary care as this afforded them greater flexibility, with care fitting around *their* daily schedule, which gave them back some control over their own lives:

*So we used to do a rota system which was so nice, because [name of private carer] would say “I’ll do morning this week” coz she was a hairdresser as well you see and she’d say “well I haven’t got anybody this week so can do every morning”, then I’d go in at dinner time and then perhaps give her tea and then [name of private carer] would go and put her to bed and we had a really good rota going (Participant 08, Daughter)*

Private carers could also carry out tasks that were out of the remit of state funded carers, which helped to lighten the informal caregivers’ load:

*She was very good, she would cook, she would do any cleaning that there was to do, she would shower him, or perhaps cut his toenails and sometimes she couldn’t do it, so she did what she could, she was very good (Participant 05, Wife)*

Private carers were often found through internet or newspaper searches. Some participants also said that they had employed trusted people from their local communities who were not trained carers. Others spoke of ‘poaching’ care staff from larger care agencies that had initially been provided to them by the local authority.

## Discussion

This study provides a detailed account of how informal caregivers’ experienced health and social care services as they cared for someone with dementia at home at the end-of-life, offering a description of the issues they encountered.

Involving families has always been a major component of palliative care philosophy and in the past two decades there has been an increased emphasis on ‘family-centred care’.^19,20^ While there has been some shift from the medical model of a hierarchical view of health care towards a more collaborative care model, findings from the current study suggest that informal caregivers looking after people with dementia at home continue to feel powerless in their role.

Caregivers often felt they had not played an active part in the decisions made for the person with dementia and there had been limited discussion about end-of-life plans, raising concerns about choice and autonomy. Whilst most caregivers had some knowledge regarding the person with dementia’s care preferences (such as a wish to stay at home), these had often not been formally documented. These findings are consistent with previous research which found that people with dementia are less likely to have any form of ACP compared to those from other disease groups and people who are cognitively intact.^21^

ACP allows individuals to identify and share their goals and preferences for future healthcare decisions. There is evidence that ACP is associated with better end-of-life outcomes including death in preferred place, satisfaction with care and treatments consistent with wishes.^22,23^ ACP’s are also reported to reduce the levels of emotional distress for families at the end of life^24^ and reduce the incidence of emergency admissions to acute care settings.^25^

Given that end-of-life decisions and preferences may change in accordance with new information or deteriorating health status,^25^ ACP should be an ongoing process, which relies on continuous input from healthcare professionals. However, caregivers reported a lack of continuity expressing confusion when navigating through services without a clear understanding of how to access support. Following their discharge from Memory Services, many did not have access to a central figure and often mentioned their need for a professional who could ‘tie things together’. They described a lack of a clear action plan and not knowing who was responsible for some aspects of care, which resulted in stress and uncertainty. Mainly this involved delays in obtaining medications and equipment at the time they were needed. The organisation of out-of-hours services at the end-of-life specifically attracted many negative comments, with reports of situations of people waiting a long time to be seen. In many cases, a lack of response could lead to bypassing out-of-hours services altogether and contacting emergency services. This could result in lengthy hospital admissions for the person with dementia and occasionally they would not return home, with them either dying in hospital or being transferred to a nursing home. Conversely, a dependence on emergency services appears to have declined since the pandemic with recent research reporting that caregivers looking after people with dementia at home at the end-of-life were reluctant to call ambulances due to a fear of hospital admission. Instead, caregivers relied upon alternative sources of support, such as charity support lines.^26^ This resonates with other research findings that caregivers were significantly less likely to call ambulances when they had 24-h access to out of hours telephone support, which also helped to reduce end-of-life hospital admissions.^27^

Poor continuity was also described in domiciliary care services with caregivers being alarmed by many different care staff attending the home. Gott et al.^28^ found that the presence of different healthcare personnel could be an ‘intrusion’ on the sense of home to older people, making them feel uncomfortable in an environment that was expected to feel familiar. Furthermore, research conducted during the pandemic found that caregivers were fearful of many different care workers entering the home due to increased risk of COVID-19 transmission.^29^ Within our study, some participants were prepared to privately fund domiciliary care to ensure they had the same, regular care worker. However, this raises concerns about the quality of services available to more deprived members of society who cannot afford to pay for private care. This reflects findings from other recent studies.^30,31^

Additionally, caregivers were often not well-informed about many aspects of domiciliary care. Many were vague about the initial assessment of their care needs, often because it took place during a time of crisis. Some felt that a process had simply ‘happened’ to them and that they had had little choice about the outcome. This was particularly notable for older participants who expressed a real fear of asking for further support in case they were deemed as being incapable and the person with dementia would be ‘taken away’ from them.

Staying silent or editing communication with health care professionals through fear of lost agency or unwanted interventions is significant given that informal caregivers are, by virtue of Government policy and campaigns by various organisations,^32,33^ entitled to be considered as ‘equal partners’ in decision making. In addition, whilst the UKs Care Act (2014)^34^ sought to give informal caregivers greater control and influence over their needs, this study demonstrates that in the context of caring for someone with dementia at home, caregivers continue to have limited power in their interactions with health care professionals. Such power disparities constitute as a barrier in attaining a collaborative relationship impeding open and honest communication.^35^ This could result in needs not being addressed, thus affecting the person with dementia’s ability to stay at home at the end-of-life.

## Considerations for practice and policy

When supporting a person with dementia at home at the end-of-life, caregivers need help in the form of hands-on care that is personalised and delivered by suitably qualified and trained professionals. As such, domiciliary care was found to be the most valuable form of support. However, years of funding reductions in social care has meant that these services are increasingly failing to deliver care that is aligned with people’s preferences.^36^

In September 2021, the UK government announced plans to reform adult social care, which included a cap on how much individuals should have to pay towards the cost of social care and a new means test. However, these plans will only apply to those starting care from October 2023 meaning that those already paying for care (or starting that process before 2023) will not benefit. Additionally, fundamental questions remain on how the reform will affect the domiciliary care workforce.

Caring as a profession is low paid and perceived as low-skill work. Due to the nature of contracts with local councils, some are forced to work under poor conditions, in which providers are paid the minimum and carers are sometimes allocated such short slots they cannot properly care for their clients. Consequently, much of the workforce is experiencing burnout.^37^ These are factors that must be considered to ensure that good quality home care is available for all in the future.

Findings also indicate that people with dementia do not formally document their wish to stay at home. National guidance advises people with dementia and their carers to engage in ACP in collaboration with health and social care professionals so that an individual’s preferences for end-of-life care are known,^12^ this includes having choice over where death occurs. As a result, future policy should lay out practical measures to enable people with dementia to make ACPs detailing place of death if they wish.

It is suggested that ACP should be initiated early in the disease trajectory, when the person still has capacity, by a trustworthy individual who is knowledgeable about their condition.^38^ Considering caregivers felt that GPs were not confident in the issues surrounding dementia, it is proposed that dementia specialists, such as Memory Services or Admiral Nurses, may be best placed to take the lead on initiating ACP. However, with the variability in follow up described above, current care arrangements for people with advanced dementia within secondary care services may not lend themselves to facilitating an appropriate, ongoing ACP process. This issue demands further consideration.

Finally, caregivers often confused the process of ACP with ‘advance directives’, which are legally binding decisions to refuse treatment. These can include a DNACPR instruction or forbid the use of ventilation or artificial means of nutrition and hydration. While most of the people with dementia had made advanced directives, these did not provide instruction on what to do when other clinical situations arose, such as the decision to use antibiotics or whether to hospitalise for conditions unrelated to dementia. So, while caregivers wanted to honour the wishes of the person with dementia by caring for them at home, they had limited guidance in determining how to do so. Decision aids which provide information on the decision and the options available have shown promise to support informal caregivers.^39,40^ These approaches may be particularly relevant during COVID-19 when decisions must be made rapidly, and there may be less support available from overstretched professionals and services.

## Strengths, limitations and further research

Our findings support and extend the knowledge provided by the limited number of previous studies from the UK and wider international literature on caring for people with dementia at home at the end-of-life. While we were not able to include extensive diversity in terms of ethnic background, participants did represent a range of ages, socioeconomic status and living circumstances. It was not the aim that the study findings be directly generalisable to other settings, but rather to provide in-depth insight into caregivers’ experiences.

Further research focussing on the perspectives of different groups of health and social care professionals to understand their experience of delivering end-of-life care in the home to people with dementia is recommended.

## Conclusions

While more people are dying at home with dementia, this increase has not been matched by an increase in resource, infrastructure, staff and capacity. Specialist palliative care for people with dementia is rare and mostly managed by GPs and domiciliary care workers. However, caregivers question their expertise in dementia and end-of-life care. Although other professional groups are involved through the illness trajectory, continuity of care was poor. Consequently, in times of crisis, caregivers often did not know who to contact for help, which could jeopardise death at home.

Domiciliary care services are failing to deliver end-of-life care that is personalised. This was reflected in accounts of inflexible services, and an over-emphasis on a task-centred approach allowing little room for negotiation and care that lacks consideration of individual wishes and circumstances. Consequently, caregivers were willing to pay high costs for private care, which inevitably restricts those who do not have the financial means to do so.

While many people with dementia had legal documents such as LPA’s and DNACPR’s in place, the formal recording of end life plans was largely absent. End-of-life care at home for people with dementia needs to be proactively planned with an emphasis on consistent ACP. Domiciliary care services also need to be formally recognised as key providers of palliative care to people with dementia in their own homes and steps must be taken to ensure staff are adequately trained and prepared to take on the responsibilities that are expected of them.

## Supplemental Material

sj-pdf-1-pmj-10.1177_02692163221092624 – Supplemental material for Health and social care services for people with dementia at home at the end of life: A qualitative study of bereaved informal caregivers’ experiences Click here for additional data file. Supplemental material, sj-pdf-1-pmj-10.1177_02692163221092624 for Health and social care services for people with dementia at home at the end of life: A qualitative study of bereaved informal caregivers’ experiences by Caroline Mogan, Karen Harrison Dening, Christopher Dowrick and Mari Lloyd-Williams in Palliative Medicine
