# Supplementary material for: Health and social care services for people with dementia at home at the end of life: A qualitative study of bereaved informal caregivers’ experiences
Source: Palliat Med. 2022 Apr 23;36(6):976–85. doi: 10.1177/02692163221092624 (PMC9174574; doi:10.1177/02692163221092624)
Supplement: sj-pdf-1-pmj-10.1177_02692163221092624 – Supplemental material for Health and social care services for people with dementia at home at the end of life: A qualitative study of bereaved informal caregivers’ experiences [file sj-pdf-1-pmj-10.1177_02692163221092624.pdf]

Supplementary File 1. Interview Prompt Guide

1. Could you start by describing you experience of looking after (insert name of person with dementia).

Professional Support

- What happened when they were diagnosed?
- What support/care/services did you/they receive when they were diagnosed?
- What support/care/services did you/they receive as the disease progressed?
- How did you find out about these services?
- How was it funded?

Informal Support

- Did you have a good informal support network around you (friends/family)?
- Who was coming to see you? What help were they giving you?
- Did you ask for help? How?
- Was it easy for you to accept help? Why?

2. When did you know that (insert name of person with dementia) was drawing close to the end of their life? What happened during this time?

Symptom management

- What were the PWD's main symptoms/problems at the end of life?
- How did you manage these? Who advised you?
- What were his/her main needs during this time?
- What types of care did you have to provide?

Equipment & Alterations to home

- What type of equipment did you have? Was it useful? Who provided/funded it?
- Did you make any alterations to your home?

3. What made you decide that you wanted to keep (insert name of person with dementia) at home during the last phase of their life?

- What types of support were offered to you and (insert name of person with dementia) during this time?
- Did you receive any funding?
- How effective was that support?
- What was good about it?
- What was not so good?

4. What were the main challenges of providing care at the end of life?

- Why do you think that it was difficult for you?

5. What helped you to cope during this time?

- Who/What was there to support you?
- Why do you think this was helpful?

6. What else could have been done to support you and (insert name of person with dementia) at the end of their life?

7. Is there anything else that you would like to add that you think might be relevant?
